# Interaction and Assembly of Bacterial Communities in High-Latitude Coral Habitat Associated Seawater

**DOI:** 10.3390/microorganisms10030558

**Published:** 2022-03-03

**Authors:** Yu Chen, Jie Li, Yuanjiao Lyu, Yiyang Zou, Qiqi Li, Qingsong Yang, Xiaoyu Tang, Xiangcheng Yuan, Zhijian Jiang, Si Zhang

**Affiliations:** 1 CAS Key Laboratory of Tropical Marine Bio-Resources and Ecology, South China Sea Institute of Oceanology, Chinese Academy of Sciences, Guangzhou 510301, China; chenyu@gmlab.ac.cn (Y.C.); yjlv_dr_em@outlook.com (Y.L.); zouyiyang18@mails.ucas.ac.cn (Y.Z.); liqiqi19@mails.ucas.ac.cn (Q.L.); qsyang@scsio.ac.cn (Q.Y.); txy506@126.com (X.T.); xcyuan@scsio.ac.cn (X.Y.); jiangzj1982@scsio.ac.cn (Z.J.); zhsimd@scsio.ac.cn (S.Z.); 2 Southern Marine Science and Engineering Guangdong Laboratory, Guangzhou 511458, China; 3 Key Laboratory of Tropical Marine Biotechnology of Hainan Province, South China Sea Institute of Oceanology, Chinese Academy of Sciences, Guangzhou 510301, China; 4 Sanya National Marine Ecosystem Research Station, South China Sea Institute of Oceanology, Chinese Academy of Sciences, Sanya 572024, China

**Keywords:** high-latitude scleractinian coral habitat, water-associated bacteria, carbon-fixation bacteria, interaction, community assembly

## Abstract

Threatened by climate change and ocean warming, coral reef ecosystems have been shifting in geographic ranges toward a higher latitude area. The water-associated microbial communities and their potential role in primary production contribution are well studied in tropical coral reefs, but poorly defined in high-latitude coral habitats to date. In this study, amplicon sequencing of 16S rRNA and *cbbL* gene, co-occurrence network, and βNTI were used. The community structure of bacterial and carbon-fixation bacterial communities showed a significant difference between the center of coral, transitional, and non-coral area. Nitrite, DOC, pH, and coral coverage ratio significantly impacted the β-diversity of bacterial and carbon-fixation communities. The interaction of heterotrophs and autotrophic carbon-fixers was more complex in the bottom than in surface water. Carbon-fixers correlated with diverse heterotrophs in surface water but fewer lineages of heterotrophic taxa in the bottom. Bacterial community assembly showed an increase by deterministic process with decrease of coral coverage in bottom water, which may correlate with the gradient of nitrite and pH in the habitat. A deterministic process dominated the assembly of carbon-fixation bacterial community in surface water, while stochastic process dominated t the bottom. In conclusion, the structure and assembly of bacterial and carbon-fixer community were affected by multi-environmental variables in high-latitude coral habitat-associated seawater.

## 1. Introduction

Coral reefs are highly diverse and productive ecosystems [1,2]. The gross primary production rates of coral reefs range from 256 to 1696 mmol C m^−2^ d^−1^ [3,4]. Benthos including coral symbiotic zooxanthellae, macroalgae, algal, turfs, and endolithic algae contribute a major fraction of the primary production in the ecosystem [5,6], while planktonic creatures contribute up to 13% of the primary production [4,5]. Moreover, environmental microbes may play a key role against reef degradation by altering its productivity and trophic dynamics [7]. Thus, environmental microbes are crucial for productivity and maintenance of coral reefs.

Benthos influences the reef water column environment at different scales [8], and bacterial communities have been found that are highly diverse and vary among reef water habitats on large scale [9]. *Alphaproteobacteria*, *Betaproteobacteria*, *Gammaproteobacteria*, and *Cyanobacteria* were high in abundance in coral reef water column [7,10]. The abundance of *Gammaproteobacteria* increased with distance away from reef, while *Alphaproteobacteria* decreased [9]. *Betaproteobacteria* was ubiquitous and did not show obvious variation in abundance across the gradient [9]. *Synechococcus*, one of the predominant carbon fixers, was found in greater abundances in the south reefs in Red Sea, where water temperature is 4 °C higher on average [11,12]. *Prochlorococcus* was more abundant in the relative oligotrophic north part of Red Sea [10,11], suggesting that different *Cyanobacteria* taxa showed diverse preference in response to temperature and nutrient supplement. Interestingly, the autotrophic carbon-fixers usually interact with heterotrophic bacteria. Such interaction had a pivotal effect on the efficiency of carbon-fixation [13], which directly influenced the primary production rate in coral reefs.

Bacterial community structure is mainly governed by deterministic processes in the ocean [14]. Many researches have focused on the coral-associated bacterial community assembly, but few on water-associated bacterial community assembly in reefs were found. In the Great Barrier Reef, seawater microbiomes were characterized by uniform community assembly patterns [15]. Similar assembly patterns were also found in cold-water coral reef ecosystems. The bacterial community was dominantly structured by the deterministic effect of microbial habitat type and the strong effect of reef location [16]. Taken together, the assembly of bacterial community in reef water was dominantly driven by deterministic effects.

Corals secrete organic-rich mucus into seawater, affecting the environmental microbial community in a continuous profile but on a small-scale [7]. Large scale surveys may ignore the effect of reef benthos, especially corals, to the seawater microbial community. Coral reefs are proposed to fix about 900 million tons of carbon per year on a global scale [17,18,19], indicating them to be potential blue carbon sinks. Therefore, it is of great importance to evaluate the effect of corals on seawater microbial community, especially the carbon-fixation community, more precisely. Over the past century, poleward migration of scleractinian corals was frequently reported due to the rising ocean surface temperature [20,21,22]. The high-latitude environments were considered to serve as climate change “refugia” for tropical coral reef species [22,23]. However, the dynamics and assembly of environmental microbiome in high-latitude scleractinian coral habitats were also unclarified, which is an obstacle to understanding the development and adaptability of high-latitude coral communities and their ecological effects. How do the water-associated bacterial and carbon-fixation bacterial community distribute in high-latitude scleractinian coral habitats? And how do they interact and assemble? To address these questions, we (1) identified the bacterial and carbon-fixation bacterial community structures in a scleractinian coral habitat in Miaowan Island, South China Sea (SCS); (2) examined the correlation of heterotrophic bacteria and autotrophic carbon-fixation bacteria; and (3) determined the assembly patterns of bacterial and carbon-fixation bacterial communities.

## 2. Materials and Methods

### 2.1. Sample Collection

**Figure 1:**
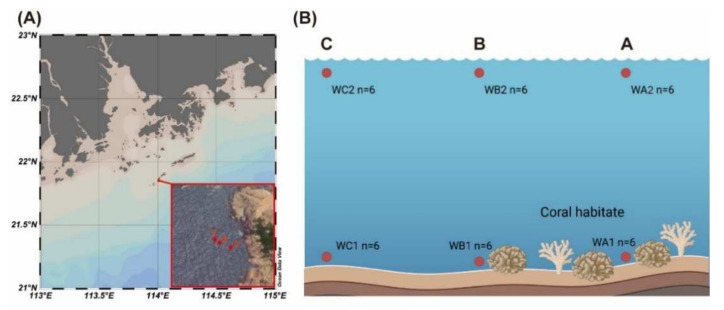
Map of the sampling stations. (**A**) Location of Miaowan Island in South China Sea and location of sampling stations. (**B**) Sampling sites of each station are in the surface and bottom of the water column. Station A is in the center of coral habitat. Station B is the marginal area of coral habitat. Station C is the non-coral area.

Miaowan Island (21°52′ N, 114°01′ E) was in the northern SCS (Figure 1A). Sampling was carried out in a small coral community at a bay of Miaowan Island. According to previous investigations, including our own, the coverage ratio of coral is 20% at the center of coral habitat (Station WA) and 5% at the marginal area (Station WB, approximately 15 m from WA). A non-coral area was selected as control (Station WC, approximately 40 m from WA). Seawater temperature was 28.1 ± 0.2 °C. Seawater samples were collected at the surface layer (0.5 m depth) and the bottom layer (*n* = 6, Figure 1B) with the assistance of a diver. Two liters of seawater was filtered onto 0.22 μm membrane filter (Millipore, Billerica, MA, USA) for DNA extraction and stored in liquid nitrogen. The map was drawn with Ocean Data View 5.4.0. The diagram of sampling sites was created with BioRender (https://biorender.com/ (accessed on 4 April 2021)).

Environmental metadata, including salinity, dissolved oxygen (DO), and pH, was recorded on board with YSI probe (YSI Pro Plus, Yellow Springs, Ohio, USA). The bottom depth of each sample site was measured using a depth sounder. The concentrations of nitrate, nitrite, ammonium, phosphate, and silicate were measured following the Chinese national standard methods GB 17378.4-2007. The DOC and DIC concentrations were analyzed using a Shimadzu TOC-L carbon analyzer. Chla was measured by acetone method after extraction with acetone for 24 h in the dark.

### 2.2. DNA Extraction, Amplification, and Sequence Analysis

Filters were cut into small pieces, and DNA was extracted using MO BIO Power Soil DNA Kit (Mo Bio, Carlsbad, CA, USA) according to the manufacturer’s instruction. Fifty nanograms of DNA was used as a template for PCR amplification. The V3V4 region of 16S rRNA genes were amplified with primers 338F and 806R [24]. PCR progress was shown as follows: 94 °C for 5 min; followed by 30 cycles of 94 °C for 30 s, 52 °C for 30 s, 72 °C for 30 s; and finished with 72 °C for 10 min. The *cbbL* gene encodes the large subunit of RubisCO type I, a key gene involved in the most distributed CO_2_-fixing pathway [25]. They were amplified with primers *cbbL*_K2f and *cbbL*_V2r [25] following the PCR progress: 94 °C for 3 min; followed by 5 cycles of 94 °C for 30 s, 45 °C for 20 s, 65 °C for 30 s; then, 20 cycles of 94 °C for 20 s, 55 °C for 20 s, 72 °C for 30 s; and finished with 72 °C for 5 min. Three replicates were pooled and purified using the QIAquick Gel Extraction Kits (QIAGEN GmbH). Product quality was assessed using a NanoDrop spectrophotometer (Thermo Scientific, Vantaa, Finland) and sequenced using an Illumina HiSeq platform with 2 × 250 bp paired-end reads for 16S rRNA gene amplicons, and Illumina MiSeq platform with 2 × 300 bp paired-end reads for *cbbL* gene amplicons.

Raw reads were quality-filtered using Fastp v0.14.1 [26] and merged using Usearch v10.0.240 [27] with a minimum overlap of five nucleotides. Maximum allowed error rate in the overlap region was 0.2. The resulting sequences were grouped by barcode using QIIME v1.9.1 [28] and were clustered as operational taxonomic units (OTUs) at 97% sequence identity using UPARSE [29]. Singleton OTUs and chimeras were removed using Usearch v10.0.240 [27] and UCHIME v4.2.40 de novo algorithm [30], respectively. OTUs were taxonomically identified using the SILVA v132 database and VSEARCH global alignments for 16S rRNA gene [31], and NCBI-NR database for *cbbL* gene. Mitochondria, chloroplasts, archaea, eukaryotes, unidentified sequences, and OTUs with abundances below 0.005% (minimum number of representative sequences) were removed [32]. The nucleotide sequences were deposited at the NCBI Sequence Read Archive under the BioProject number PRJNA762627.

### 2.3. Statistical Analysis

Alpha diversity indices including Richness, Chao1, Shannon, and Simpson index were calculated with Picante [33] package in R. Non-metric multidimensional scaling (NMDS) analysis by Bray–Curtis distance was applied for illustrating the compositions of total 16S rRNA and *cbbL* OTUs in all groups. The analysis of similarities (ANOSIM), redundancy analysis (RDA), and Mantel test were calculated with Vegan [34] package in R. Construction of Neighbor-joining phylogenetic trees (bootstraps = 1000) for top 70 *cbbL* OTU sequences was performed with megaX [35]. Key OTUs for bacterial communities were selected by Random Forest machine learning with the randomForest [36] package in R. Co-occurrence network of carbon fixers and heterotrophs in surface or bottom water (*n* = 18) were computed using the CoNet (v1.1.1.beta) plugin within Cytoscape (v3.7.2). β-mean-nearest taxa distance (βMNTD) was calculated with Picante [33] package in R. To generate the null distribution, random shuffling of OTU labels across the tips of the phylogeny was calculated 999 times. βNTI was calculated to represent the difference between observed βMNTD and the mean of the null distribution (|βNTI| > 2: deterministic; |βNTI| < 2: stochastic) [37].

## 3. Results

### 3.1. Alpha Diversity of Bacteria and Carbon-Fixation Bacteria

For 16S rRNA gene sequencing, a total of 3,162,205 reads were recovered from sequencing of the mixture of PCR amplicons of the pooled DNA. OTUs were defined at a sequence similarity cut-off of 97% using average linkage hierarchical clustering. In surface seawater, the Richness and Chao1 indices of 16S rRNA genes were significantly higher in non-coral station (WC2) than in coral-associated areas (WA2 and WB2). The Shannon and Simpson indices were similar. In bottom seawater, the Richness, Chao1, and Shannon indices of 16S rRNA genes were significantly higher in non-coral station (WC1), while the Simpson index was similar (Figure S1).

For *cbbL* gene sequencing, 6,442,176 reads were obtained. OTUs were also defined at 97% of sequence similarity. Alpha diversity of *cbbL* genes was similar to the 16S rRNA genes in all stations. In surface seawater, the Richness and Chao1 indices of *cbbL* genes were significantly higher in non-coral station (WC2). In bottom seawater, the Richness, Chao1, and Shannon indices of *cbbL* genes were also significantly higher in non-coral station (WC1, Figure S2). Simpson index did not show a significant difference in surface or bottom water.

### 3.2. Composition of Bacterial and Carbon-Fixation Bacterial Community

**Figure 2:**
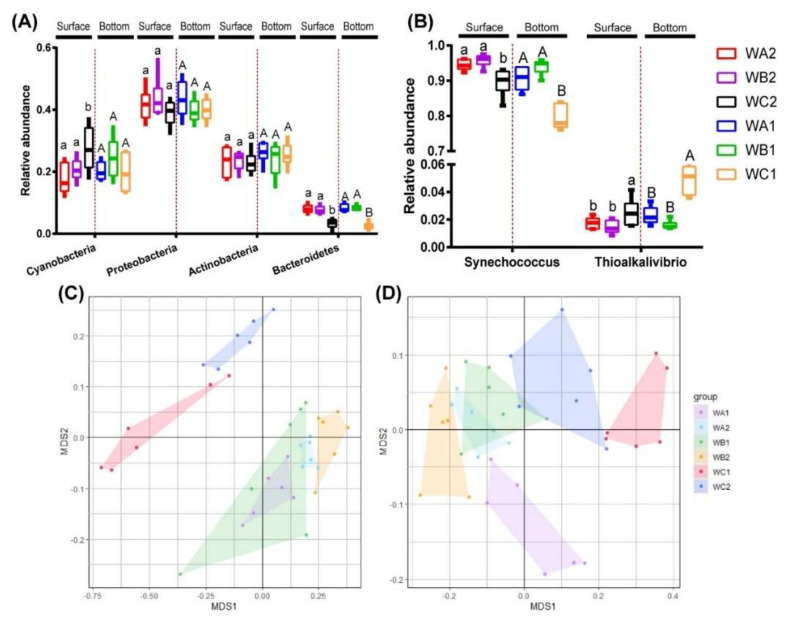
Relative abundance of key taxa and beta diversity of bacterial and carbon-fixation bacterial community. (**A**) Variation in relative abundance of bacterial phylum (>5%) between areas. (**B**) Variation in relative abundance of carbon-fixation bacterial genus (>1%) between areas. NMDS ordinations based on Bray–Curtis dissimilarity shows the compositions of total 16S rRNA (**C**) and *cbbL* (**D**) gene OTUs.

In total bacterial community, *Proteobacteria* (37.8–43.7%), *Actinobacteria* (23.2–26.6%), *Cyanobacteria* (17.7–27.8%), and *Bacteroidetes* (4.6–9.0%) were the dominant groups (Figure S3). In surface water, the relative abundance of *Cyanobacteria* was significantly higher, while *Bacteroidetes* was significantly lower in the non-coral station (WC2) than coral-associated stations (WA2 and WB2, One-way ANOVA *p* < 0.05). The relative abundance of *Proteobacteria* and *Actinobacteria* did not show significant difference in all sites. In bottom water, only the relative abundance of *Bacteroidetes* was found significantly lower in non-coral station (WC1) than coral-associated stations (WA1 and WB1, One-way ANOVA *p* < 0.05), while the abundance of *Proteobacteria*, *Actinobacteria*, and *Cyanobacteria* was unchanged among all sites (Figure 2A).

In carbon-fixation communities, *Synechococcus* was the predominant genus, with relative abundance that ranged from 76.1% to 97.5% (Figure S3). Its relative abundance was significantly higher in coral-associated stations (WA1/WA2/WB1/WB2) than in non-coral stations (WC1/WC2, One-way ANOVA *p* < 0.05) in both surface and bottom water. *Thioalkalivibrio* was the second most abundant genus, whose relative abundance ranged from 1.2 to 5.8%. It was significantly more abundant in non-coral stations (WC1/WC2, Figure 2B).

Distribution specificity of bacteria was assessed by comparing the similarities of communities. NMDS based on Bray–Curtis dissimilarities revealed a clear separation of the bacterial communities from different stations (Figure 2C). Such specificity was further confirmed by ANOSIM analysis. In surface water, the bacterial communities of WA2 and WB2 did not show significant difference (R = 0.1287, *p* = 0.108). However, bacterial community of WC2 significantly varied from WA2 (R = 0.8611, *p* = 0.003) and WB2 (R = 0.4667, *p* = 0.005). In bottom water, communities of three stations varied significantly (WA1/WB1, R = 0.3315, *p* = 0.006; WA1/WC1, R = 0.7046, *p* = 0.003; WB1/WC1, R = 0.8148, *p* = 0.001).

Carbon-fixation communities were also separately revealed by Bray–Curtis dissimilarity-based NMDS analysis (Figure 2D). Similar to the bacterial communities, carbon-fixation communities of WA2 and WB2 did not vary significantly (R = 0.1333, *p* = 0.096), while WC2 showed significant difference in surface water (WA2/WC2, R = 0.8741, *p* = 0.003; WB2/WC2, R = 0.4833, *p* = 0.004). In bottom water, communities of three stations varied significantly (WA1/WB1, R = 0.3574, *p* = 0.008; WA1/WC1, R = 0.7167, *p* = 0.003; WB1/WC1, R = 0.8481, *p* = 0.003).

### 3.3. Relationship between Microbial Communities and Environment

**Figure 3:**
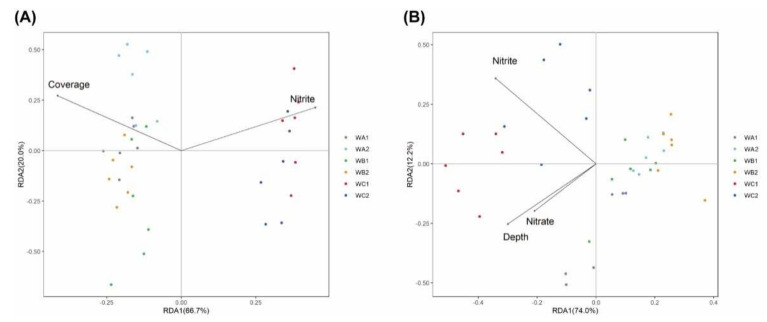
Ordination diagrams from redundancy analysis (RDA) of 16S rRNA (**A**) and *cbbL* (**B**) communities and environmental parameters (*n* = 6). Arrows indicate the direction and magnitude of environmental variables associated with 16S rRNA and *cbbL* community structures. Each sample is represented by a colored circle.

The relationship of microbial communities and environmental metadata was analyzed via transformation-based redundancy analysis (tb-RDA) and partial Mantel tests, respectively. Environmental metadata including water depth (Depth); concentration of nitrite, ammonium, nitrate, phosphate, silicate, and chlorophyll a (Chla); DOC; DIC; salinity (Sal); pH; and coral coverage ratio (Coverage) were analyzed. For bacterial community, stepwise forward selection indicated that nitrite concentration and coral coverage ratio were the best predicators, explaining 18% (axis 1: 66.7%, axis 2: 20.0% after stepwise forward selection) of the total variables. Coral coverage ratio was positively correlated with β-diversity of bacterial community at coral-associated stations (WA and WB), while concentration of nitrite was positively correlated with that at non-coral station (WC, Figure 3A). Partial Mantel tests indicated that coral coverage ratio was significantly correlated with changes in bacterial communities in both surface and bottom water, while concentrations of DOC and pH were significantly correlated with bacterial community changing in surface water (Table S1).

For carbon-fixation communities, tb-RDA with stepwise forward selection indicated water depth and concentration of nitrate, and nitrite explained 23% (axis 1: 74.0%, axis 2: 12.2% after stepwise forward selection) of the total variables. These parameters were negatively correlated with β-diversity of the community at coral-associated stations (WA and WB), but positively correlated with non-coral station (WC, Figure 3B). Partial Mantel tests show that coral coverage ratio was significantly correlated with changing of carbon-fixation communities in both surface and bottom water, while concentration of nitrite and pH were significantly correlated in surface water (Table S2).

### 3.4. Phylogenetic Diversity of Carbon-Fixation Bacteria

**Figure 4:**
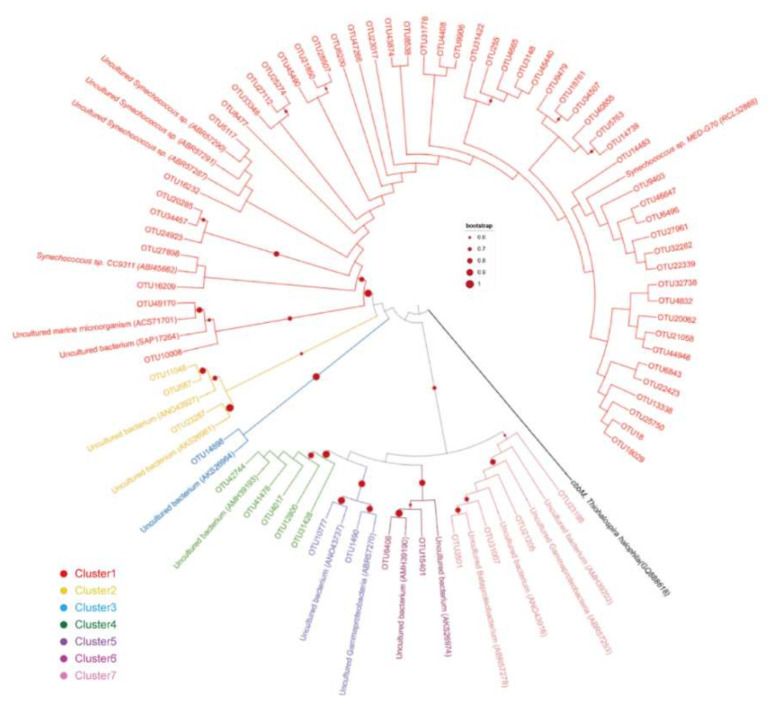
Neighbor-joining tree obtained from deduced amino acid sequences of representative *cbbL* OTUs, together with reference sequences retrieved from the NCBI database. The OTUs with relative abundance over 0.001 were selected for phylogenetic analysis and these OTUs were manually divided into seven clusters based on sequence similarity. The *cbbM* gene from *Thiohalospira halophila* is used as out group. One thousand bootstrap replicates were performed. Nodes with bootstrap value over 0.6 were marked.

Phylogenetic analysis was conducted with *cbbL* gene OTUs with relative abundance > 0.1%. Seventy representative OTUs were retrieved and their deduced amino acid sequences were used for Neighbor-joining tree construction (Figure 4). Accordingly, these OTUs were manually divided into seven clusters based on bootstrap values (bootstrap > 0.6). Cluster 1 was the most abundant group (82.1%), with OTUs affiliating to *Synechoccocus*. They clustered with two known *Synechoccocus* species and five uncultured microorganisms. Clusters two to seven all grouped with uncultured microorganisms (Figure 4). Cluster two contained OTUs affiliating to *Thioalkalivibrio*, with relative abundance of 0.4%. Cluster three contained one OUT affiliating to *Chromatiales*, with relative abundance of 0.1%. OTUs from Cluster four affiliated to *Gammaproteobacteria*, with relative abundance of 1.0% in total. Cluster five and six contained OTUs affiliating to *Chromatiaceae*, with relative abundance of 0.3% and 0.6%, respectively. Cluster seven contained OTUs affiliating to *Chromatiales*, with relative abundance of 0.9%.

### 3.5. Interaction of Key Bacteria and Carbon-Fixers

**Figure 5:**
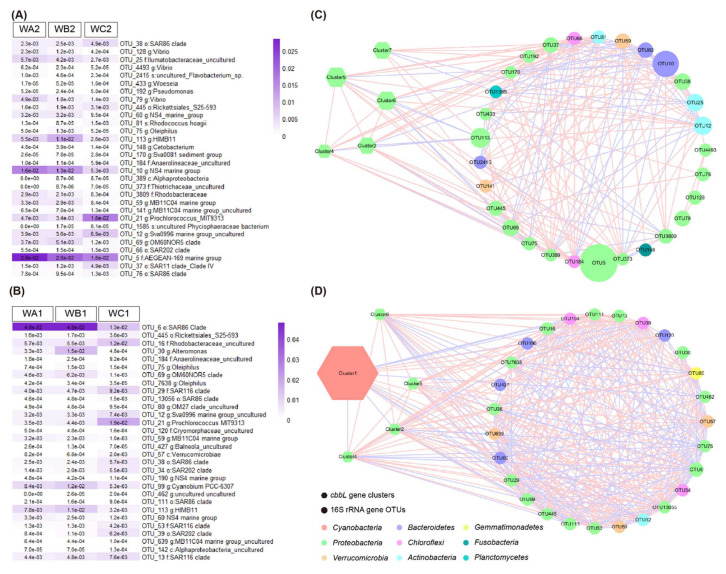
Correlation of key 16S rRNA gene OTUs and *cbbL* gene clusters. (**A**,**B**) Abundance of top 30 key 16S rRNA gene OTUs in surface (**A**) and bottom water (**B**) selected by Random Forest machine learning. (**C**,**D**) The co-occurrence network of key 16S rRNA gene OTUs and *cbbL* gene clusters in surface water (**C**) and bottom water (**D**). Node size indicated the abundance of the OTU.

A total of 30 key 16S rRNA gene OTUs were selected via Random Forest analysis for surface (Figure 5A) and bottom (Figure 5B) water, respectively. The key OTUs largely belonged to heterotrophic bacteria. Co-occurrence network of key 16S rRNA gene OTUs and the *cbbL* gene clusters was more complex in bottom water than in the surface (Figure 5C,D). A total of 369 edges were present in the network for bottom water, while 186 edges in surface water. In surface water, five clusters were correlated with the key OTUs. Cluster 2 positively correlated with two OTUs from *Actinobacteria*, two from *Chloroflexi* and four from *Proteobacteria*, while it negatively correlated with one OTU from *Bacteroidetes* and three from *Proteobacteria*. Cluster four positively correlated with one OTU from *Actinobacteria*, two from *Chloroflexi*, and three from *Proteobacteria*, while it negatively correlated with two from *Proteobacteria*. Cluster five positively correlated with two OTUs from *Actinobacteria*, two from *Chloroflexi*, and four from *Proteobacteria*, but negatively correlated with one from *Bacteroidetes* and *Proteobacteria*, respectively. Cluster six positively correlated with two OTUs from *Actinobacteria*, *Chloroflexi*, and *Proteobacteria* respectively, while it negatively correlated with one OTU from *Bacteroidetes* and three from *Proteobacteria*. Cluster seven positively correlated with two OTUs from *Bacteroidetes*, two from *Verrucomicrobia*, and one from *Proteobacteria*, while it negatively correlated with one OTU from *Planctomycetes* (Figure 5C, Table S3).

In bottom water, five of the *cbbL* gene clusters co-occurred with key OTUs, but the clusters were different from those in surface water. Cluster one positively correlated with three OTUs from *Bacteroidetes*, two from *Proteobacteria*, and three from *Verrucomicrobia*, while it negatively correlated with one OTU from *Actinobacteria*, two from *Chloroflexi*, and five from *Proteobacteria*. Cluster two, four, five, and six showed similar profiles in correlation with key OTUs. They all positively correlated with one OTU from *Actinobacteria*, three from *Chloroflexi*, eight from *Proteobacteria*, one from *Verrucomicrobia*, and one from *Gemmatimonadetes*, while it negatively correlated with *Bacteroidetes*, *Proteobacteria*, and *Verrucomicrobia* taxa (Figure 5D, Table S4).

### 3.6. Assembly of Bacterial and Carbon-Fixation Bacterial Communities

**Figure 6:**
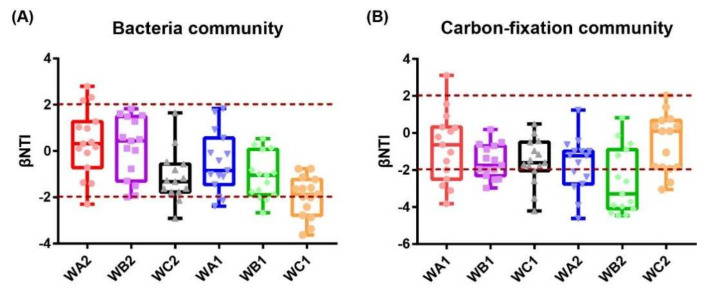
Distribution patterns of βNTI values. (**A**) Bacterial communities in surface and bottom water. (**B**) Carbon-fixation bacterial communities in surface and bottom water. Horizontal dashed lines indicate lower and upper significance thresholds at −2 and +2, respectively.

The βNTI for each station were calculated. For bacterial community, 26.7% and 13.3% of the absolute βNTI values were >2 in WA2 and WC2 station in the surface, and none of the βNTI values fell in this area at WB2. While in bottom water, 13.3%, 20%, and 40% of the absolute βNTI values were >2 in WA1, WB1, and WC1, respectively. In addition, the proportion showed an increase along with distance to coral community (Figure 6A), suggesting that bacterial community was dominantly assembled with stochastic pattern in surface water, while the environmental impacts increased on community assembly with distance to center of coral habitat in the bottom. For carbon-fixation community, the proportions of absolute βNTI values >2 were 40%, 66.7%, and 20.0% in WA2, WB2, and WC2 in the surface, respectively. In the bottom, 33.3%, 26.7%, and 26.7% of absolute βNTI values were >2 in WA1, WB1, and WC1, respectively (Figure 6B), indicating that assembly of the carbon-fixation bacterial community was dominated by a deterministic process in surface water, but dominated by a stochastic process at the bottom.

Partial mantel test was conducted to evaluate the correlation of environmental parameters with variation in βNTI distribution. Coral coverage was the most important factor that correlated with both bacterial and carbon-fixation community in the surface and bottom. Nitrite concentration and pH were also correlated with both communities in surface water. Concentration of silicate and phosphate were correlated with bacterial and carbon-fixation community, respectively (Tables S5 and S6).

## 4. Discussion

Previous studies identified the water-associated microbial community structure in large scale surveys. In this study, we surveyed a high-latitude scleractinian coral community, providing more precise clues for interaction and assembly of bacterial and carbon-fixation bacterial community in water column.

### 4.1. Environmental Drivers of the Bacterial Distribution in Coral-Associated Water Column

Microbial community composition is closely related to the physical and chemical features of this habitat, including temperature, salinity, nutrients, and the hydrological regime [10]. Generally, bacterial community of inshore and other eutrophic reef waters with high nutrient concentrations comprised 37–73% of total biomass in heterotrophic microbes [38,39], dominated by *Bacteroidetes* and *Proteobacteria*, especially *Gammaproteobacteria* [40,41,42]. In this study, *Proteobacteria*, *Actinobacteria*, *Cyanobacteria*, and *Bacteroidetes* were dominant phylum in all samples, which was consistent with most previous studies. *Synechococcus* was the predominant autotrophic bacteria in eutrophic reef waters, and they usually comprised more than 90% of *Cyanobacteria* in tropical coastal ocean waters [39,43,44,45]. The nutrient level of seawater at Miaowan Island changed seasonally due to Pearl River runoff and human behavior [46]. Our study found that the concentrations of nitrate, ammonia, and phosphate were 0.29 mg/L, 0.022 mg/L, and 0.003 mg/L (Table S7), respectively, indicating a eutrophic environment. So, *Synechococcus* were unsurprisingly the predominant autotrophs here.

The diversity of bacterial and carbon-fixation communities showed trends to increase when area shifting from coral-associated (WA and WB) to non-coral (WC). Moreover, community structures of bacteria and carbon-fixation bacteria were significantly different between coral-associated (WA and WB) and non-coral (WC) areas in surface and bottom water, indicating the distribution-specificity of communities. Similar shifting in bacterial community was also found in a cold-water coral reef ecosystem where the community structure changed significantly from reef center to the periphery in a small-scale, suggesting a significant biogeographic imprinting of seawater-associated community [16]. However, the dynamic of carbon-fixation community had not yet been identified before. Taken together, our results shed light on the dynamic of bacterial and carbon-fixer communities in water of a high-latitude scleractinian coral habitat, which indicates its sensitivity and correlation to the benthic community.

Furthermore, our results demonstrated that coral coverage ratio, nitrite, and nitrate significantly influence the bacterial and carbon-fixation bacterial community. Nitrogen supplement was one of the limitations for bacterial growth and productivity [47,48]. The symbionts of scleractinian coral were found to be able to fix nitrogen and nitride-enriched mucus was released into the seawater by corals, which is an essential nutrient for environmental microbes [49,50,51,52]. Additionally, the sediment is considered to be another nitrogen source in some coral reefs. The flux rates of inorganic nitrogen from the sediment to the water column were about 0.046 nmol cm^−2^ h^−1^ [53]. Taken together, nitrogen supplement associated with coral community may be the key environmental factor that impacts the water bacterial and carbon-fixation bacterial community. These results highlight that investigating the microbiome and their interactions in three environments, including the sediment, coral, and water, in an integrated manner, is critical for understanding the health and resilience of coral reefs as a single functioning ecosystem.

### 4.2. Interactions between Heterotrophic Bacteria and Carbon-Fixation Bacteria

Despite the environmental factors, the correlation of biotic factors within the microbial community is taken into consideration. Interactions between heterotrophic and photoautotrophic microbes are essential in the marine food web [54,55]. Heterotrophs utilize the organic carbon fixed by autotrophs. In return, they benefit photoautotrophs by providing essential micronutrients, such as vitamins, bioavailable trace metals, and amino acids [55,56,57], as well as reducing the levels of toxic reactive oxygen [58,59]. The co-culture system proved that the interaction between strictly heterotrophic bacteria and autotrophic strains played a crucial role in improving the CO_2_ fixing efficiency by eliminating self-restraint of organic compounds and promoting the autotrophic pathway [13]. Moreover, such interaction was usually changed by temperature, seawater pH, and nutrient supplement [60,61,62,63]. Thus, the interactions between heterotrophic bacteria and carbon-fixers are essential for microbial community assembly. However, most of the researches were performed in the lab. Few clues were obtained from in situ environmental researches.

In this study, we analyzed the interaction of carbon-fixers and key bacteria. The key bacteria in the communities were largely identified as heterotrophs, making it possible to access their interactions in situ. The complexity of network in bottom water was more than on the surface. Moreover, the carbon-fixation taxa clusters were more connective to the heterotrophic bacteria in the bottom, suggesting a more interactive community there. Interestingly, the correlation pattern between carbon-fixation taxa correlated and heterotrophs showed evident difference in surface and bottom water. The clusters in the surface correlated with different heterotrophic taxa. However, in bottom water, clusters interacted with fewer lineages of heterotrophic taxa, except for the Cluster 1. SAR86, SAR11, and SAR202 were ubiquitous heterotrophs interacting with autotrophic clusters affiliated to *Proteobacteria* in the bottom water. These heterotrophic taxa are dominant bacteria in the ocean and usually co-occur with the autotrophs [64,65,66,67]. They mediate fundamental ecological processes, for example driving the geochemical cycles [64,65,66]. Our results indicate that *Synechococcus* (except for Cluster 1) may be in support with these key heterotrophs in our studying field.

On the other hand, the most abundant Cluster 1 affiliated to *Synechococcus* interacted with *Bacteroidetes*, *Proteobacteria*, and *Verrucomicrobia* taxa, suggesting they may be functionally correlated with carbon cycle in bottom water of high-latitude coral habitat. Additionally, Cluster 1 was found to interact with three taxa from *Flavobacteriales*, which were reported to be in need for rapid growth of *Synechococcus* [68]. Heterotrophic bacteria were able to help with the formation of picocyanobacteria aggregate, which was essential for the adaptation of these small cells living in subsurface water [69]. Taken together, most carbon-fixers (abundance > 84.7%) closely interacted with heterotrophic bacteria in the bottom, suggesting more connective but less diverse interaction, as well as a more complex food and energy network at the bottom of scleractinian coral habitat. The debate on the role of coral reefs as a carbon source or sink never came to an end [19,70]. Our results provided some preliminary clues, at least in this high-latitude habitat, that carbon-fixers were more abundant in the center of coral habitat. In addition, they were more connective with heterotrophs as closer to the coral colonies in the bottom, indicating that corals may promote the carbon-fixation activity by seawater microbiome.

### 4.3. Assembly of Environmental Bacterial and Carbon-Fixation Bacterial Community in Seawater of High-Latitude Coral Habitat

Microbial communities are governed by deterministic and stochastic processes, and both of them regulated the assembly of microbial community concurrently [71]. The assembly patterns of microbial community in large-scale coral reefs were well defined. In the Great Barrier Reef, the composition of the microbial community showed significantly higher similarity ‘within’ than ‘between’ samples collected from the same habitat but different time, suggesting that deterministic rather than stochastic processes drive their community assembly [15]. In another case, the assembly of microbial community in cold-water coral reef ecosystems was driven by both stochastic and deterministic processes [16]. Moreover, variable selection was identified to be the main factor determining the compositional change in the bacterial community in coral reefs of Red Sea [72]. However, the assembly pattern remained unclarified in high-latitude coral habitats.

In our study, we identified the assembly pattern of bacterial and carbon-fixation bacterial communities in seawater of a high-latitude scleractinian coral habitat. For bacterial community, deterministic and stochastic processes together drove the community assembly in surface water in the coral-associated (WA2) and non-coral (WC2) areas, but only stochastic processes existed in the transitional area (WB2). Interestingly, in the bottom water, the proportion of deterministic process gradually increased from the coral-associated area (WA1) to non-coral area (WC1), suggesting an increased impact of environmental factors on bacterial community assembly with a distance away from the center of coral habitat. This may due to the high primary production ratio of the coral community and increased nutrient supplement by corals [7], which induced lower competition among bacteria [73]. The concentration of nitrite and pH showed significant correlation between OTU abundance and βNTI in surface water, suggesting they may be the key factors driving the assembly of bacterial community.

The assembly pattern of carbon-fixation bacterial community was poorly defined. Here in this study, we found that deterministic and stochastic processes co-existed, and the proportions were discrepant between stations, suggesting that assembly of carbon-fixation bacterial community was affected by environmental factors with varying degrees. Community assembly was dominated by the deterministic process in surface water, while stochastic process dominated at the bottom. Nitrite and pH were strongly correlated with βNTI in surface water, implicating that the assembly of carbon-fixation community was mainly driven by these parameters. However, phosphate was the only chemical parameter with weak correlation with βNTI in bottom water, suggesting that there may be other undetermined environmental factors. Therefore, our results revealed the impacts of environmental conditions on assembly of bacterial and carbon-fixation bacterial communities in high-latitude coral habitat seawater, which may advance the understanding of formation and sustainment of coral reef at high-latitude area due to global warming.

## 5. Conclusions

In this study, we investigated the diversity of bacterial and carbon-fixation community in seawater of a high-latitude coral habitat in the northern SCS and revealed that the compositions of both communities significantly changed between different areas in the habitat. Heterotrophic bacteria were more connective with carbon-fixers in bottom water than in surface water, and the autotrophs tended to interact with fewer lineages of heterotrophic taxa in bottom water. The bacterial community assembly showed an increase by deterministic process with a decrease of coral coverage in bottom water, which may correlate with the gradient of nitrite concentration and pH in the habitat. The assembly of carbon-fixation communities did not show variation between different areas but water layers. The deterministic process was dominant in surface water, while the stochastic process was dominant at the bottom.

## Data Availability

The nucleotide sequences were deposited at the NCBI Sequence Read Archive under the BioProject number PRJNA762627.

## Supplementary Materials

The following supporting information can be downloaded at: https://www.mdpi.com/article/10.3390/microorganisms10030558/s1, Figure S1: Alpha diversity indices of bacterial community; Figure S2: Alpha diversity indices of carbon-fixation microbial community; Figure S3: Composition of bacterial and carbon-fixation microbial community; Table S1: Correlation of environmental variables with bacterial OTU abundance determined by partial Mantel tests; Table S2: Correlation of environmental variables with *cbbL* gene OTU abundance determined by partial Mantel tests; Table S3: Correlation of key 16S rRNA gene OTUs with *cbbL* gene clusters in surface water; Table S4: Correlation of key 16S rRNA gene OTUs with *cbbL* gene clusters in bottom water; Table S5: Correlation of environmental variables with βNTI values of bacterial community determined by partial Mantel tests; Table S6: Correlation of environmental variables with βNTI values of carbon-fixation microbial community determined by partial Mantel tests; Table S7: Environmental characters of all sampling sites.
