# Supplementary material for: Interaction and Assembly of Bacterial Communities in High-Latitude Coral Habitat Associated Seawater
Source: Microorganisms. 2022 Mar 3;10(3):558. doi: 10.3390/microorganisms10030558 (PMC8955259; doi:10.3390/microorganisms10030558)
Supplement: Supplementary file 1 [file microorganisms-10-00558-s001.zip › microorganisms-1593637-supplementary.pdf]

microorganisms

MDPI

Supplementary Material

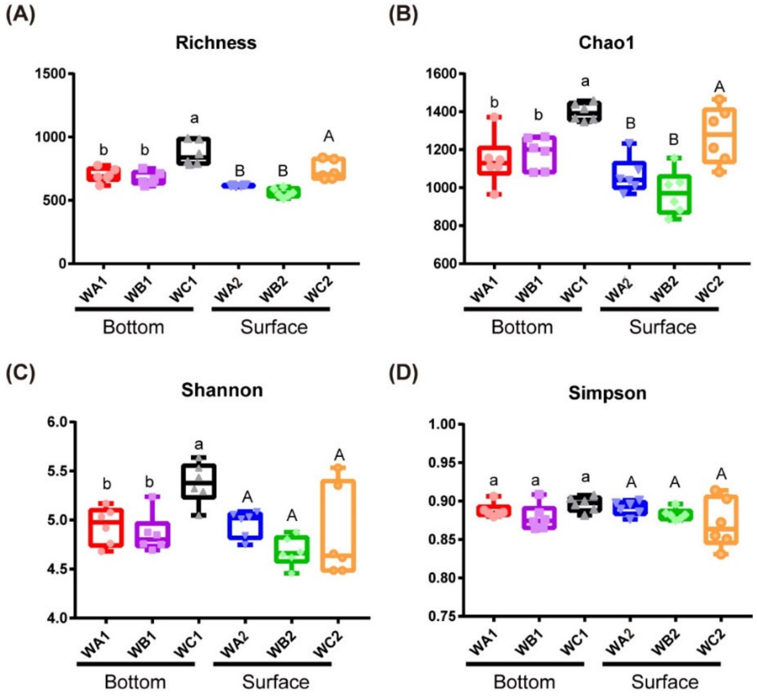

Figure S1. Alpha diversity indices of bacterial community. Different letters indicated the statistical significance (One-way ANOVA, p<0.05).

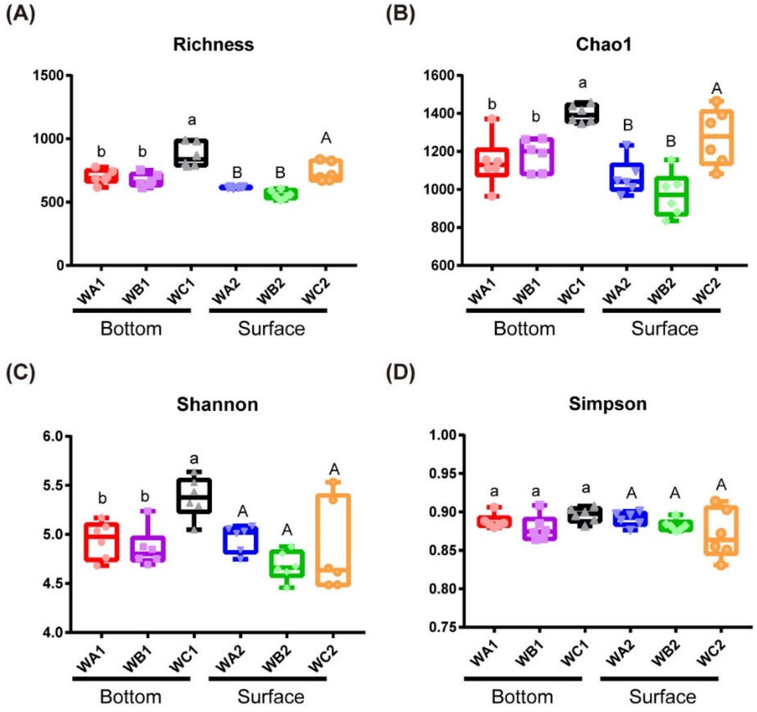

Figure S2. Alpha diversity indices of carbon-fixation microbial community. Different letters indicated the statistical significance (One-way ANOVA, p<0.05).

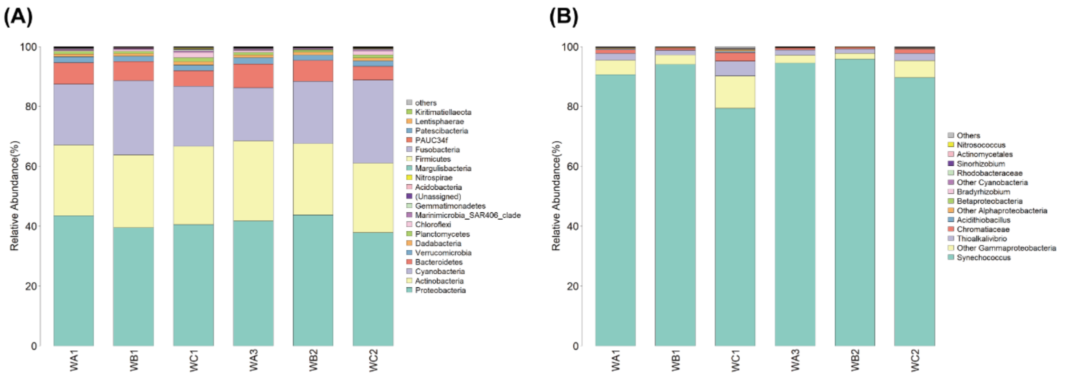

Figure S3. Composition of bacterial and carbon-fixation microbial community. (A) Bacterial phyla with top 20 in abundance were shown. (B) Relative abundance of carbon-fixation microbial taxa.

Table S1. Correlation of environmental variables with bacterial OTU abundance determined by partial Mantel tests (Permutations: 9999).

	Surface water		Bottom water	
	**R value**	***p* value**	**R value**	***p* value**
Nitrite	0.150	0.088	0.199	0.060
Ammonium	0.054	0.320	-0.127	0.838
Nitrate	0.192	0.065	0.044	0.365
Phosphate	0.165	0.124	-0.078	0.690
Silicate	-0.045	0.640	0.202	0.085
DOC	0.270	0.017	-0.113	0.808
Salinity	0.112	0.160	-0.063	0.680
pH	0.520	0.001	0.095	0.157
DIC	-0.155	0.880	0.156	0.126
Coverage	0.406	0.001	0.319	0.001
Chlorophyll a	-0.032	0.560	0.007	0.258
Depth	ND	ND	0.058	0.281

DOC, dissolved organic carbon; DIC, dissolved inorganic carbon; ND, not determined.

Table S2. Correlation of environmental variables with carbon-fixation microbial OTU abundance determined by partial Mantel tests (Permutations: 9999).

	Surface water		Bottom water	
	**R value**	**p value**	**R value**	**p value**
Nitrite	0.371	0.003	0.182	0.100
Ammonium	0.059	0.280	-0.090	0.702
Nitrate	0.022	0.377	-0.018	0.490
Phosphate	0.155	0.164	0.148	0.161
Silicate	0.198	0.053	-0.070	0.633
DOC	-0.093	0.730	-0.203	0.980
Salinity	-0.172	0.930	0.024	0.371
pH	0.477	0.001	-0.003	0.460
DIC	-0.020	0.496	0.003	0.445
Coverage	0.301	0.003	0.306	0.007
Chlorophyll a	-0.080	0.650	-0.053	0.641
Depth	ND	ND	0.063	0.244

DOC, dissolved organic carbon; DIC, dissolved inorganic carbon; ND, not determined.

Table S3. Correlation of key 16S rRNA gene OTUs with *cbbL* gene clusters in surface water.

		**Cluster2**	**Cluster4**	**Cluster5**	**Cluster6**	**Cluster7**
Positive	*Actinobacteria*	2 | OTU81; OTU12	1 | OTU81	2 | OTU81; OTU12	2 | OTU81; OTU12	
	Bacteroidetes					2 | OTU60; OTU10
	Chloroflexi	2 | OTU66; OTU184	2 | OTU66; OTU184	2 | OTU66; OTU184	2 | OTU66; OTU184	
	Proteobacteria	4 | OTU445; OTU389; OTU373; OTU37	3 | OTU389; OTU373; OTU37	4 | OTU389; OTU373; OTU37; OTU192	2 | OTU373; OTU37	1 | OTU113
	Verrucomicrobia					2 | OTU59; OTU141
Negative	*Bacteroidetes*	1 | OTU60		1 | OTU10	1 | OTU60	
	Proteobacteria	3 | OTU75; OTU69; OTU113	2 | OTU75; OTU113	1 | OTU113	3 | OTU75; OTU69; OTU113	
	Planctomycetes					1 | OTU1585

Table S4. Correlation of key 16S rRNA gene OTUs with *cbbL* gene clusters in bottom water.

		**Cluster1**	**Cluster2**	**Cluster4**	**Cluster5**	**Cluster6**
Positive	*Actinobacteria*		1 | OTU12	1 | OTU12	1 | OTU12	1 | OTU12
	Bacteroidetes	3 | OTU60; OTU190; OTU120				
	Chloroflexi		3 | OTU39; OTU34; OTU184	3 | OTU39; OTU34; OTU184	3 | OTU39; OTU34; OTU184	3 | OTU39; OTU34; OTU184
	Proteobacteria	2 | OTU7638; OTU6	8 | OTU53; OTU445; OTU38; OTU29; OTU16; OTU13056; OTU13; OTU111	8 | OTU53; OTU445; OTU38; OTU29; OTU16; OTU13056; OTU13; OTU111	8 | OTU53; OTU445; OTU38; OTU29; OTU16; OTU13056; OTU13; OTU111	8 | OTU53; OTU445; OTU38; OTU29; OTU16; OTU13056; OTU13; OTU111
	Verrucomicrobia	3 | OTU69; OTU639; OTU59	1 | OTU57	1 | OTU57	1 | OTU57	1 | OTU57
	Gemmatimonadetes		1 | OTU80	1 | OTU80	1 | OTU80	1 | OTU80
Negative	*Actinobacteria*	1 | OTU12				
	Bacteroidetes		1 | OTU60	1 | OTU60		1 | OTU60
	Chloroflexi	2 | OTU39; OTU184				
	Proteobacteria	5 | OTU53; OTU462; OTU29; OTU16; OTU111	4 | OTU75; OTU69; OTU6; OTU113	4 | OTU75; OTU7638; OTU6; OTU113	4 | OTU75; OTU69; OTU6; OTU113	4 | OTU75; OTU69; OTU6; OTU113
	Verrucomicrobia		1 | OTU639	1 | OTU639		2 | OTU639; OTU59

Table S5. Correlation of environmental variables with βNTI values of bacterial community determined by partial Mantel tests (Permutations: 9999).

	**Surface water**		**Bottom water**	
	**R value**	***p* value**	**R value**	***p* value**
Nitrite	0.243	0.008	0.153	0.118
Ammonium	-0.004	0.503	-0.003	0.472
Nitrate	0.079	0.234	-0.012	0.506
Phosphate	-0.071	0.710	-0.085	0.704
Silicate	0.070	0.236	0.390	0.005
DOC	0.099	0.183	-0.027	0.556
Salinity	-0.069	0.727	-0.099	0.808
pH	0.327	0.001	0.104	0.130
DIC	0.031	0.394	0.074	0.277
Coverage	0.307	0.001	0.364	0.001
Chlorophyll a	0.121	0.160	0.049	0.302
Depth	ND	ND	0.099	0.170

Table S6. Correlation of environmental variables with βNTI values of carbon-fixation microbial community determined by partial Mantel tests (Permutations: 9999).

	**Surface water**		**Bottom water**	
	**R value**	***p* value**	**R value**	***p* value**
Nitrite	0.443	<0.001	0.134	0.085
Ammonium	-0.127	0.862	-0.045	0.664
Nitrate	0.098	0.215	0.103	0.134
Phosphate	0.294	0.026	0.183	0.039
Silicate	-0.014	0.516	0.034	0.376
DOC	0.096	0.223	-0.164	0.956
Salinity	-0.049	0.630	0.168	0.058
pH	0.556	<0.001	0.044	0.269
DIC	-0.095	0.738	0.085	0.187
Coverage	0.255	0.010	0.478	0.001
Chlorophyll a	-0.059	0.649	0.058	0.238
Depth	ND	ND	0.125	0.087

Table S7. Environmental characters of all sampling sites.

Samples	Nitrite (mg/L)	Ammonium (mg/L)	Nitrate (mg/L)	Phosphate (mg/L)	Silicate (mg/L)	DOC (mg/L)	Depth (m)	DIC (mg/L)	Chla (mg/m^3)	Salinity	pH
WA1.1	0.0022	0.018	0.032	0.002	0.303	1.446	9.3	25.45	0.5601	33.74	7.78
WA1.2	0.0017	0.014	0.033	0.003	0.345	1.404	7.9	25.74	0.3231	33.73	7.77
WA1.3	0.0022	0.03	0.027	0.001	0.165	1.314	7.3	25.51	0.7817	33.79	7.79
WA1.4	0.002	0.022	0.034	0.001	0.183	1.431	8.8	25.17	0.4416	33.71	7.83
WA1.5	0.002	0.022	0.046	0.003	0.195	1.376	10.2	25.56	0.5601	33.67	7.82
WA1.6	0.0011	0.017	0.027	0.003	0.093	1.493	9.9	25.29	0.5763	33.68	7.83
WA2.1	0.0017	0.02	0.028	0.002	0.129	1.456	0.5	25.37	0.3231	33.76	7.84
WA2.2	0.0022	0.053	0.037	0.002	0.171	1.523	0.5	25.57	0.4416	33.84	7.83
WA2.3	0.0025	0.027	0.02	0.002	0.225	1.499	0.5	25.41	0.3231	33.86	7.83
WA2.4	0.0031	0.026	0.032	0.001	0.189	1.575	0.5	25.14	0.1185	33.73	7.82
WA2.5	0.0028	0.033	0.034	0.002	0.183	1.877	0.5	25.54	0.1185	33.75	7.81
WA2.6	0.0022	0.028	0.03	0.003	0.153	1.333	0.5	25.43	0.1185	33.76	7.82
WB1.1	0.002	0.028	0.017	0.003	0.129	1.474	11.7	24.99	0.5439	33.84	7.87
WB1.2	0.0022	0.014	0.028	0.002	0.153	1.379	12.9	25.41	0.3231	33.86	7.89
WB1.3	0.0028	0.017	0.027	0.002	0.189	1.566	13.8	25.34	0.2208	33.86	7.88
WB1.4	0.0014	0.012	0.032	0.003	0.213	1.363	13.2	25.22	0.7809	33.71	7.88
WB1.5	0.002	0.013	0.019	0.002	0.225	1.421	12.7	25.17	0.3393	33.72	7.89
WB1.6	0.0031	0.025	0.02	0.002	0.201	1.351	11.9	25.49	0.3231	33.75	7.91
WB2.1	0.002	0.018	0.025	0.003	0.201	1.273	0.5	24.74	1.0878	33.75	7.8
WB2.2	0.0028	0.024	0.015	0.002	0.159	1.271	0.5	24.99	0.8832	33.79	7.81
WB2.3	0.0017	0.013	0.028	0.002	0.093	1.391	0.5	26.1	0.1023	33.77	7.81
WB2.4	0.0025	0.03	0.023	0.002	0.225	1.371	0.5	24.11	0.2208	33.7	7.84
WB2.5	0.002	0.014	0.031	0.003	0.141	1.341	0.5	25.58	0.2208	33.76	7.85
WB2.6	0.002	0.017	0.02	0.002	0.153	1.312	0.5	25.84	0.867	33.87	7.84
WC1.1	0.0025	0.015	0.034	0.003	0.225	1.436	11	25.29	0.1185	33.74	7.78
WC1.2	0.0043	0.039	0.03	0.004	0.171	1.42	14.6	25.5	0.1185	33.77	7.83
WC1.3	0.0034	0.019	0.031	0.003	0.225	1.456	12.3	25.43	0.1185	33.83	7.81
WC1.4	0.0028	0.011	0.023	0.002	0.267	1.431	13.2	25.24	0.1185	33.76	7.87
WC1.5	0.0034	0.017	0.024	0.007	0.195	1.394	14.5	25.15	0.1185	33.96	7.88
WC1.6	0.0031	0.012	0.037	0.006	0.183	1.348	13.9	26.02	0.3393	33.89	7.89
WC2.1	0.0028	0.011	0.053	0.003	0.087	1.494	0.5	25.26	0.1185	33.7	7.87
WC2.2	0.0034	0.015	0.024	0.007	0.165	1.417	0.5	25.26	0.2208	33.79	7.87
WC2.3	0.0034	0.036	0.02	0.002	0.189	1.466	0.5	25.21	0.4416	33.74	7.87
WC2.4	0.0025	0.017	0.028	0.004	0.237	1.453	0.5	26.42	0.3231	33.7	7.9
WC2.5	0.0034	0.013	0.034	0.002	0.207	1.47	0.5	25.73	0.1185	33.89	7.92
WC2.6	0.004	0.016	0.038	0.004	0.105	1.395	0.5	25.39	0.2208	33.74	7.92
